# The Fokker–Planck equation of the superstatistical fractional Brownian motion with application to passive tracers inside cytoplasm

**DOI:** 10.1098/rsos.221141

**Published:** 2022-11-02

**Authors:** C. Runfola, S. Vitali, G. Pagnini

**Affiliations:** ^1^ Department of Physics and Astronomy, University of Bologna, Viale Berti Pichat 6/2, I-40127 Bologna, Italy; ^2^ BCAM – Basque Center for Applied Mathematics, Alameda de Mazarredo 14, E-48009 Bilbao, Basque Country, Spain; ^3^ Eurecat, Centre Tecnológic de Catalunya, Unit of Digital Health, Data Analytics in Medicine, E-08005 Barcelona, Catalunya, Spain; ^4^ Ikerbasque – Basque Foundation for Science, Plaza Euskadi 5, E-48009 Bilbao, Basque Country, Spain

**Keywords:** anomalous diffusion, Fokker–Planck equation, Erdélyi–Kober fractional equation, Krätzel function, mRNA molecules, *Escherichia coli* cells

## Abstract

By collecting from literature data experimental evidence of anomalous diffusion of passive tracers inside cytoplasm, and in particular of subdiffusion of mRNA molecules inside live *Escherichia coli* cells, we obtain the probability density function of molecules’ displacement and we derive the corresponding Fokker–Planck equation. Molecules’ distribution emerges to be related to the Krätzel function and its Fokker–Planck equation to be a fractional diffusion equation in the Erdélyi–Kober sense. The irreducibility of the derived Fokker–Planck equation to those of other literature models is also discussed.

## 1. Introduction

The experimental evidence of anomalous diffusion in living systems has been definitively established [1–3], and in particular, we recall the measurements of the motion of mRNA molecules inside live *Escherichia coli* cells by Golding and Cox [4], which are now a milestone in the field. Here, we derive the Fokker–Planck equation for the probability density function (PDF) of molecules’ displacement in agreement with the main findings from such dataset and similar findings.

Unfortunately, the PDF of passive tracers in cytoplasm is not available yet from data because of technical issues that limit the number of particles' trajectories, and this affects, in particular, the reliability of the tails of the distribution, which are the footprint of deviation from Gaussianity and standard diffusion. Thus, in these circumstances with lack of measurements, we do not analyse experimental data, but we collect experimental results [4–11] to derive first the molecules’ PDF and then the corresponding Fokker–Planck equation. If the solution, namely, the PDF, is already known, then the governing equation can be considered useless, but, in the following, we discuss how indeed knowing the PDF is not all in the game, and the knowledge of the corresponding equation provides indeed further valuable information.

In general, similar features of anomalous diffusion have been experimentally observed in the motion of different passive tracers in different living systems (see e.g. [12–18]), and the fractional Brownian motion (fBm) emerged to be the underlying random motion of molecules’ trajectories (see e.g. [19–25]), as well as the generalized Gamma distribution with its special cases emerged to be the distribution of the diffusion coefficients (e.g. [18,26,27]). Therefore, a superstatistical fBm resulted to be a successful model [10,24,28].

The superstatistical fBm is indeed a randomly scaled Gaussian process that was studied for fractional anomalous diffusion originally within the framework of the generalized grey Brownian motion (ggBm) [28–32], which recently has been extended to investigate the relation with generalized time-fractional diffusion equations [33,34]. Moreover, randomly scaled Gaussian processes resembling the ggBm have been formulated for modelling and understanding anomalous diffusion also within an under-damped approach [35–37].

The qualitative success of the superstatistical fBm for modelling anomalous diffusion in living systems was already discussed within the framework of the ggBm in relation to ergodicity breaking and fractional diffusion [28]. The PDF of molecules is therefore a mixture of Gaussian distributions with random variances, and within a Bayesian approach, such population can be understood as the likelihood modulated by the prior distribution of a parameter. The formal randomization of this parameter is equivalent to the computation of the marginal likelihood, which corresponds indeed to the PDF of independent and identically distributed (i.i.d.) random variables [38]. This view allows for highlighting and clarifying the role of the central limit theorem in the dynamics of an heterogeneous ensemble of Brownian particles as in the superstatistical fBm here considered [38]. Any value of the variance of the Gaussian PDFs is idiosyncratic, and the random diffusion coefficients represent the heterogeneity of the ensemble of molecules, which is sufficient to (weakly) break the ergodicity of the system [28].

To conclude, we report that the superstatical modelling of anomalous diffusion can be related also with the effect of probes’ polydispersity that generates an apparent anomalous diffusion as emerged in the cytoplasm of human cells where the apparent anomaly exponent decreases with increasing polydispersity of the probes [39]. These results can be applied also in intracellular studies of the mobility of nanoparticles, polymers or oligomerizing proteins.

Here, we found that the PDF of molecule displacement belongs to the family of the Krätzel special function [40], and its Fokker–Planck equation is a fractional diffusion equation in the Erdélyi–Kober sense [41,42] that cannot be reduced to existing models for anomalous diffusion.

The rest of this article is organized as follows. In §2, we present the dataset by Golding and Cox [4] and the related findings from the corresponding data analysis. In §3, we establish the molecule PDF, and in §4, we derive the governing Fokker–Planck equation. In §5, we discuss the results and in particular the valuable information behind the determination of the Fokker–Planck equation in spite of the known PDF. Section 6 is devoted to the conclusions and future perspectives.

## 2. The paradigmatic dataset by Golding and Cox

In this study, we are interested in the governing equation of the PDF of passive tracers diffusing in cytoplasm, because of the information that it can give about the process (see §5), apart from the PDF itself that is its fundamental solution. In particular, for our aims, we mainly consider the results obtained from the data acquired by Golding and Cox [4]. The number of trajectories included in that dataset is 21, and so they are not enough for calculating ensemble averages. In this respect, we recall here that anomalous diffusion is indeed characterized also by a non-Gaussian distribution of particles and therefore also by the scaling of the tails of the distribution that are indeed affected by the size of the sample of the observations.

In their experiment, they considered the motion of mRNA molecules released from their template DNA and free to move in the cytoplasm, and in particular, they tracked the random motion of individual fluorescently labelled mRNA molecules inside live *E. coli* cells. This dataset turned out to be a benchmark dataset for studying anomalous diffusion in living systems and was widely analysed in these years (see e.g. [5–11]). The main findings from the Golding and Cox experiment [4] can be summarized as follows.

Let $X_{t}$ be the molecule position at time *t* > 0, the time-averaged mean square displacement (TA-MSD) of molecules resulted to be subdiffusive $\bar{{\left[X_{t+\Delta }-X_{t}\right]}^{2}}∼\Delta^{\beta }$ [4], with *β* ∈ (0, 1). Moreover, TA-MSD emerged to be quite scattered [4,10,11], and this can be due to a population of diffusion coefficients that leads to weak ergodicity breaking [18,28].

Furthermore, the application of the method of *p*-variation showed that the underlying motion of the molecules is more likely the fBm [5,6,10]. Therefore, by adopting a superstatistics of fBm, namely, by adopting the process $X_{t}=\sqrt{\Lambda }\;B_{t}^{H}$, where Λ is a non-negative random variable and $B_{t}^{H}$ is the fBm with Hurst exponent *H* ∈ (0, 1), it was possible to estimate the diffusion coefficients of the molecules’ ensemble, which resulted in a population related to the Weibull distribution [10], which actually is a special case of the generalized Gamma distribution. The ggBm is recovered when Λ is a totally skewed positive *α*-stable random variable with stable index *α* ∈ (0, 1) [28,29]. A Weibull distribution of the diffusion coefficients has been confirmed [11] also when the fBm was replaced by the so-called fractional Lévy stable motion (FLSM) that is a Lévy-driven stochastic process that generalizes the fBm [7,11].

Trajectories from the Golding–Cox dataset are not enough for properly testing mixing and ergodicity by comparing ensemble and time averages. However, necessary conditions for mixing and ergodicity can be derived on the basis of a single trajectory [9]. Trajectories from the Golding–Cox dataset satisfy the necessary conditions for mixing and ergodicity [9], which is consistent with both the fBm [10] and the proper FLSM model [8,9]. Actually, if each trajectory is properly re-scaled, then their scattering is largely reduced and the ergodicity breaking is no longer present [10].

Since the FLSM is based on Lévy stable distributions, diverging statistics, e.g. the MSD, are replaced by the sample MSD that may exhibit either normal or anomalous diffusion. The FLSM results to perform comparable or even better than the fBm, at least for some of the trajectories, for what concerns some observable as the Hurst exponent, stability index and both sample MSD and sample *p*-variation [7]. This may be due to the fact that the experimental trajectories may deviate from Gaussianity, contrary to the fBm approach, and then the more flexible FLSM allows for catching this feature [11].

Actually, further valuable experimental data on diffusion of passive tracers in cytoplasm have been published and also their analysis, beside those related with the Golding–Cox dataset [4]. Here, we recall, for example, the studies of particle motion in crowded fluids, which is an analogue of the motion inside the cytoplasm of living cells, by using dextran dissolved in water [43–45] or purely viscous solution obtained by using sucrose into water [45] that led to the understanding of the corresponding diffusive motions in terms of the fBm [20,22], as well as the measurements of the dynamics of histone-like nucleoid-structuring proteins in live *E. coli* [46], where a power-law distribution of the diffusion coefficients of individual proteins has been observed in agreement with the Pearson type VII distribution that led to the development of a modelling approach based on the fBm that hierarchically takes into account the joint fluctuations of both the anomalous diffusion exponents and the diffusion constants [24], or diffusion of tracer particles in the cytoplasm of mammalian cells where the experimental observations are described by an intermittent fBm alternating between two states of different mobility [22] and similarly in the case of intracellular endosomes [23], suggesting that the underlying trajectories can be modelled by a fBm with a distributed Hurst exponent [25].

## 3. Superstatistical molecules’ distribution

In the case under consideration, the PDF of molecules results to be given by the superstatistical integral

3.1 $$P(x;t)=\int_{0}^{\infty }G(x;t|\lambda )f\;(\lambda )\;d\lambda ,$$

where

3.2 $$G(x;t|\lambda )=\frac{1}{\sqrt{4\pi \lambda t^{2H}}}\;e^{-x^{2}/(4\lambda t^{2H})}$$

is the Guassian PDF of the fBm and *f*(*λ*) is, in short notation, the generalized Gamma distribution

**Figure 1.**
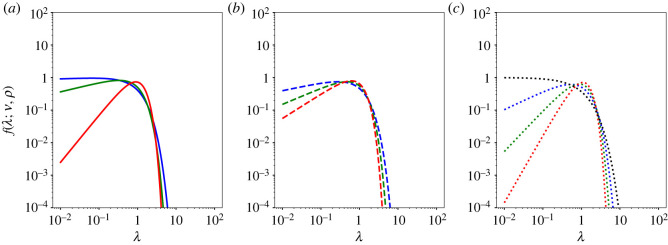
Plots of the distribution of the diffusion coefficients *f*(*λ*) (3.3) for different values of the parameters: the generalized Gamma distribution (*ν* = 1.25 and *ρ* = 1.5 (solid blue line), *ν* = 1.5 and *ρ* = 1.75 (solid green line), *ν* = 1.75 and *ρ* = 1.25 (solid red line)); the Weibull distribution (*ν* = *ρ* = 1.25 (dashed blue line), *ν* = *ρ* = 1.5 (dashed green line), *ν* = *ρ* = 1.75 (dashed red line)); the Gamma distribution (*ρ* = 1) (*ν* = 1.25 (dotted blue line), *ν* = 1.5 (dotted green line), *ν* = 1.75 (dotted red line)) and the exponential distribution (*ρ* = *ν* = 1 (dotted black line)). In all the plots, *λ*_0_ = 1.

3.3 $$f\;(\lambda )=f\;(\lambda ;\lambda_{0},\nu ,\rho )=\frac{\rho }{\lambda_{0}^{\nu }\;\Gamma (\nu /\rho )}\lambda^{\nu -1}e^{-{\left(\lambda /\lambda_{0}\right)}^{\rho }},$$

with *λ*, *λ*_0_, *ν*, *ρ* > 0. The generalized Gamma distribution reduces to the Weibull distribution when *ν* = *ρ*, i.e. *f*(*λ*; *λ*_0_, *ρ*, *ρ*) = *W*(*λ*; *λ*_0_, *ρ*), to the Gamma distribution when *ρ* = 1 and to the exponential distribution when *ρ* = *ν* = 1; see a comparison shown in figure 1.

The generalized Gamma distribution was studied in the framework of superstatistics in both the original formulation [47] and the recent formulations for anomalous diffusion within the diffusing diffusivity [48,49] and the ggBm approaches [48].

We observe that molecules’ PDF (3.1) can be rewritten as follows:

3.4 $$P(x;t)=\frac{\rho }{\Gamma (\nu /\rho )}\frac{1}{\sqrt{4\pi \lambda_{0}t^{2H}}}\;Z_{\rho }^{\nu -1/2}\left(\frac{x^{2}}{4\lambda_{0}t^{2H}}\right),$$

where $Z_{\rho }^{\nu }(\cdot )$ is the Krätzel function [40]

3.5 $$Z_{\rho }^{\nu }(u)=\int_{0}^{\infty }\lambda^{\nu -1}e^{-(u/\lambda )-\lambda^{\rho }}\;d\lambda ,\;u>0,$$

and the variance of particle displacement is expressed as follows:

3.6 $$\begin{array}{rl} \left\langle x^{2}\right\rangle & =2\int_{0}^{\infty }x^{2}P(x;t)\;dx\\ & =\int_{0}^{\infty }2\lambda t^{2H}\;f\;(\lambda )\;d\lambda \\ & =2\langle \lambda \rangle t^{2H},\;\mathrm{with}\;\langle \lambda \rangle =\int_{0}^{\infty }\lambda f\;(\lambda )\;d\lambda =\lambda_{0}\;\frac{\Gamma [(\nu +1)/\rho ]}{\Gamma (\nu /\rho )}. \end{array}$$

**Figure 2.**
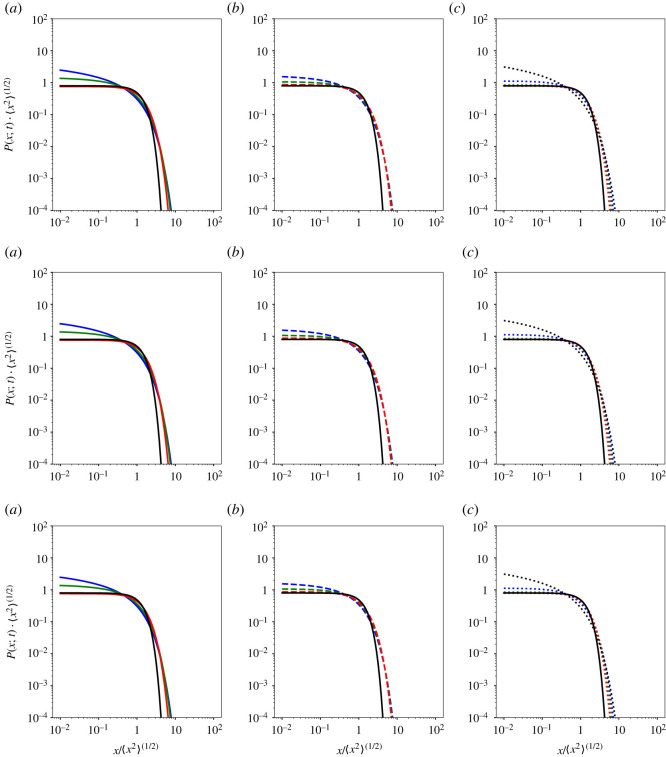
Plots of the molecules’ PDF (3.1), i.e. 〈*x*^2^〉^1/2^ *P*(*x*; *t*) vs *x*/〈*x*^2^〉^1/2^, in lin-log scale with the following values of the parameters of the distribution of the diffusion coefficients *f*(*λ*) (3.3): the generalized Gamma distribution (*ν* = 1.25 and *ρ* = 1.5 (solid blue line), *ν* = 1.5 and *ρ* = 1.75 (solid green line), *ν* = 1.75 and *ρ* = 1.25 (solid red line)); Weibull distribution with (*ν* = *ρ* = 1.25 (dashed blue line), *ν* = *ρ* = 1.5 (dashed green line), *ν* = *ρ* = 1.75 (dashed red line)); the Gamma distribution (*ρ* = 1) (*ν* = 1.25 (dotted blue line), *ν* = 1.5 (dotted green line), *ν* = 1.75 (dotted red line)); and the exponential distribution (*ρ* = *ν* = 1 (dotted black line)). In all the plots, *λ*_0_ = 1. The reference with the Gaussian density is also displayed (solid black line). (*a*): *H* = 0.25; (*b*): *H* = 0.5; (*c*): *H* = 0.75; where any difference due to *H* is indeed removed because of the self-similarity with respect to the variance.

Plots of the molecules’ PDF (3.1) are shown in figure 2.

Moreover, we consider now the Mellin transform pair [50]

3.7 $$M_{r}\{\varphi ;s\}=\int_{0}^{\infty }\varphi (r)\;r^{s-1}\;dr$$

and

3.8 $$\varphi (r)=\frac{1}{2\pi i}\int_{L}M_{r}\{\varphi ;s\}\;r^{-s}\;ds,$$

where L is a specific contour path that separates the poles that provide through the residue theorem a power-series with positive exponents from those poles that provide a power-series with negative exponents. Thus, we obtain the following Mellin–Barnes integral representation:

3.9 $$P(x;t)=\frac{1}{\Gamma (\nu /\rho )}\frac{1}{\sqrt{4\pi \lambda_{0}t^{2H}}}\frac{1}{2\pi i}\int_{L}\Gamma (s)\Gamma \left(\frac{s+\nu }{\rho }-\frac{1}{2\rho }\right){\left[\frac{x^{2}}{4\lambda_{0}t^{2H}}\right]}^{-s}\;ds.$$

For mathematical convenience, we introduce now the change of variable *u* = *x*^2^/[4*λ*_0_ *t*^2*H*^], and from the normalization constraint ∫P(x;t) dx=∫P(u) du=1, we have

3.10 $$P(u)=\frac{\rho }{\Gamma (\nu /\rho )}\frac{1}{\sqrt{4\pi u}}\;Z_{\rho }^{\nu -\frac{1}{2}}(u).$$

By using formula (3.9), the representation in terms of the H-Fox function [40,41] of the PDF (3.10) results to be

3.11 $$P(u)=\frac{1}{\Gamma (\nu /\rho )}\frac{1}{\sqrt{4\pi }}H_{0,2}^{2,0}\left[u|\begin{array}{c} -\\ \left(-1/2,1),((\nu -1)/\rho ,1/\rho \right) \end{array}\right],$$

and by considering the properties of Mellin–Barnes integrals [50], and so the properties of the H-Fox functions as well [51], we can obtain from (3.9) the following asymptotic expansions for *u* → 0:

3.12 $$P(u)≃\frac{1}{\Gamma (\nu /\rho )}\frac{1}{\sqrt{4\pi }}\;O(u^{d}),$$

with *d* = min [ − 1/2, *ν* − 1], and for *u* → +∞,

3.13 $$P(u)≃\frac{1}{\Gamma (\nu /\rho )}\frac{1}{\sqrt{4\pi }}\;O\left(u^{\left(\nu -\rho -1)/(\rho +1\right)}\exp\left[-(\rho +1){\left(\frac{u}{\rho }\right)}^{\rho /(\rho +1)}\right]\right).$$

## 4. Derivation of the Fokker–Planck equation

The problem to derive the Fokker–Planck equation of (3.1) is similar to the problem to derive the relation between generalized diffusion equations and subordination schemes [52]. Here, to derive the Fokker–Planck equation governing PDF (3.1), we first consider the following diffusion equation solved by the Gaussian PDF of the fBm (3.2):

4.1 $$\frac{\partial G}{\partial t}=2\lambda Ht^{2H-1}\frac{\partial^{2}G}{\partial x^{2}},$$

and, by multiplying both sides times *f*(*λ*) and integrating over *λ*, from formula (3.1), we have

4.2 $$\begin{array}{rl} \frac{\partial }{\partial t}P(x;t,\nu ,\rho ) & =2Ht^{2H-1}\frac{\partial^{2}}{\partial x^{2}}\int_{0}^{\infty }\lambda G(x;t|\lambda )f\;(\lambda )\;d\lambda \\ & =2H\lambda_{0}t^{2H-1}\frac{\partial^{2}}{\partial x^{2}}\left[\frac{\Gamma ((\nu +1)/\rho )}{\Gamma (\nu /\rho )}P(x;t,\nu +1,\rho )\right], \end{array}$$

where the extended notation *P*(*x*; *t*, *ν*, *ρ*) for PDF (3.1) is used for highlighting the difference between the PDFs from both sides of the equation.

By using formula (3.9), we obtain

4.3 $$\begin{array}{rl} P(x;t,\nu +a,\rho ) & =\frac{\Gamma (\nu /\rho )}{\Gamma ((\nu +a)/\rho )}\frac{1}{2\pi i}\int_{L}\frac{\Gamma (((\nu +a)/\rho )-(s/2H\rho ))}{\Gamma (\nu /\rho -(s/2H\rho ))}M_{t}\{P(x;t,\nu ,\rho );s\}\;t^{-s}\;ds\\ & =\frac{\Gamma (\nu /\rho )}{\Gamma ((\nu +1)/\rho )}{\;}_{t}D_{2H\rho }^{\nu /\rho -1,a/\rho }P(x;t,\nu ,\rho ), \end{array}$$

where ${\;}_{t}D_{\eta }^{\gamma ,\mu }$, with *μ*, *η* > 0 and γ∈R, is the Erdélyi–Kober fractional operator with respect to *t* ([41], formula (3.14)),

4.4 $${\;}_{t}D_{\eta }^{\gamma ,\mu }\varphi (t)=\frac{1}{2\pi i}\int_{L}\frac{\Gamma (1+\gamma +\mu -(s/\eta ))}{\Gamma (1+\gamma -(s/\eta ))}M_{t}\{\varphi ;s\}\;t^{-s}\;ds.$$

Therefore, by plugging (4.3) with *a* = 1 into (4.2), we have that (4.2) is indeed a fractional diffusion equation in the Erdélyi–Kober sense,

4.5 $$\frac{\partial P}{\partial t}=2H\lambda_{0}t^{2H-1}{\;}_{t}D_{2H\rho }^{\nu /\rho -1,1/\rho }\frac{\partial^{2}P}{\partial x^{2}}.$$

As an alternative to this derivation based on the superstatistical fBm, another statistical perspective for the emerging of Erdélyi–Kober fractional calculus comes from generalizations of entropy [42,53,54]. Moreover, for general fractional differential equations in the Erdélyi–Kober sense, approximations and numerical schemes are also available [55–57].

Finally, with reference to the Golding and Cox dataset [4], since the diffusion coefficients follow a Weibull distribution [10], i.e. *ν* = *ρ*, the generalized Fokker–Planck equation (4.5) turns into

4.6 $$\frac{\partial P}{\partial t}=2H\lambda_{0}t^{2H-1}{\;}_{t}D_{2H\rho }^{0,1/\rho }\frac{\partial^{2}P}{\partial x^{2}},$$

where 2*H* = 0.70 ± 0.07 [4], $\lambda_{0}^{1/2}=0.06$ and 2*ρ* = 1.84 [10]. In fact, if the adopted superstatistical notation is $X_{t}=Y\;B_{t}^{H}$ [10], then variable *Y* results to be distributed according to the Weibull distribution $W(y;\lambda_{0}^{1/2},2\rho )$. This does not affect any formula, but it must be accounted for when a comparison with empirical data is performed.

As a concluding remark, we compare the Fokker–Planck equation (4.5), or its special case (4.6), against other equations used in anomalous diffusion. From formula (4.4), the following noteworthy identities can be derived [41]:

4.7 $${\;}_{t}D_{1}^{0,\mu }\varphi (t)=t^{-\mu }{{\;}_{t}D}_{1}^{-\mu ,\mu }[t^{\mu }\varphi (t)]={{\;}_{t}D}_{\mathrm{RL}}^{\mu }[t^{\mu }\varphi (t)],\;\mu >0,$$

where ${\;}_{t}D_{\mathrm{RL}}^{\mu }$ is the fractional derivative in the Riemann–Liouville sense [41], and we observe that formula (4.7) highlights the relation between Erdélyi–Kober fractional equations and Riemann–Liouville (or Caputo with proper initial conditions) fractional differential equations with time-varying coefficients [58]. Thus, when *ν* = *ρ* = 1/(2*H*), equations (4.5) and (4.6) become

4.8 $$\frac{\partial P}{\partial t}=2H\lambda_{0}t^{2H-1}{\;}_{t}D_{\mathrm{RL}}^{2H}\left[t^{2H}\frac{\partial^{2}P}{\partial x^{2}}\right],$$

which is different from existing fractional diffusion models with time-dependent diffusion coefficient [58–61], and moreover, it cannot be reduced further up to the so-called time-fractional diffusion equation,

4.9 $$\frac{\partial \varphi }{\partial t}=\lambda_{0}{\;}_{t}D_{\mathrm{RL}}^{1-2H}\frac{\partial^{2}\varphi }{\partial x^{2}}.$$

Equation (4.9) is the fractional Fokker–Planck equation, for example, of a continuous-time random walk (CTRW) with a fat-tailed distribution of waiting times (see e.g. [62]), but also for the intermediate asymptotic regime of a CTRW with two Markovian hopping-trap mechanisms [63]. Such CTRW models have been used for modelling anomalous diffusion in living systems (see e.g. subdiffusion of lipid granules in living fission yeast cells [17], and also see e.g. [64–66]), for explaining some statistical features that appear also in the Golding and Cox dataset [4].

The failure of the CTRW approach for modelling some common features of anomalous diffusion in living systems was already pointed out [5,6,18], and an alternative and promising approach seemed to be the ggBm [10,28]. As a matter of fact, the generalized Fokker–Planck equation for the ggBm emerged to be, as well, a fractional diffusion equation in the Erdélyi–Kober sense as follows [67]:

4.10 $$\frac{\partial \varphi }{\partial t}=\lambda_{0}\;2H\rho \;t^{2H-1}{\;}_{t}D_{2H\rho }^{1/\rho -1,1-1/\rho }\frac{\partial^{2}\varphi }{\partial x^{2}},$$

but it is not related neither to (4.5) nor to (4.6) in any one of their special cases. On the contrary, from (4.7) with *ρ* = 1/(2*H*), equation (4.10) reduces to (4.9), such that the governing equation of the CTRW is indeed a special case of the governing equation of the ggBm.

The fundamental solution of (4.10) is provided by plugging in (3.1), in place of the generalized Gamma distribution (3.3), the following population of diffusion coefficients:

4.11 $$f_{\mathrm{ggBm}}(\lambda )=\frac{1}{\lambda_{0}}M_{1/\rho }\left(\frac{\lambda }{\lambda_{0}}\right)=\frac{1}{\lambda_{0}}\sum_{n=0}^{\infty }\frac{{\left(-\lambda /\lambda_{0}\right)}^{n}}{n!\Gamma [-n/\rho +(1-1/\rho )]},$$

with 0 < 1/*ρ* < 1, where $M_{\beta }(z)$ is the M-Wright Mainardi function [68,69], and as a matter of fact, the fundamental solution of (4.10) here denoted by *P*_ggBm_(*x*; *t*) is also an M-function [29,67], i.e.

4.12 $$P_{\mathrm{ggBm}}(x;t)=\frac{1}{2}\frac{1}{\sqrt{\lambda_{0}t^{2H}}}M_{1/(2\rho )}\left(\frac{\left|x\right|}{\sqrt{\lambda_{0}t^{2H}}}\right),$$

and then by setting *ρ* = 1/(2*H*), we have also the fundamental solution of (4.9) that is here denoted by *P*_CTRW_(*x*; *t*), i.e.

4.13 $$P_{\mathrm{CTRW}}(x;t)=\frac{1}{2}\frac{1}{\sqrt{\lambda_{0}t^{2H}}}M_{H}\left(\frac{\left|x\right|}{\sqrt{\lambda_{0}t^{2H}}}\right).$$

**Figure 3.**
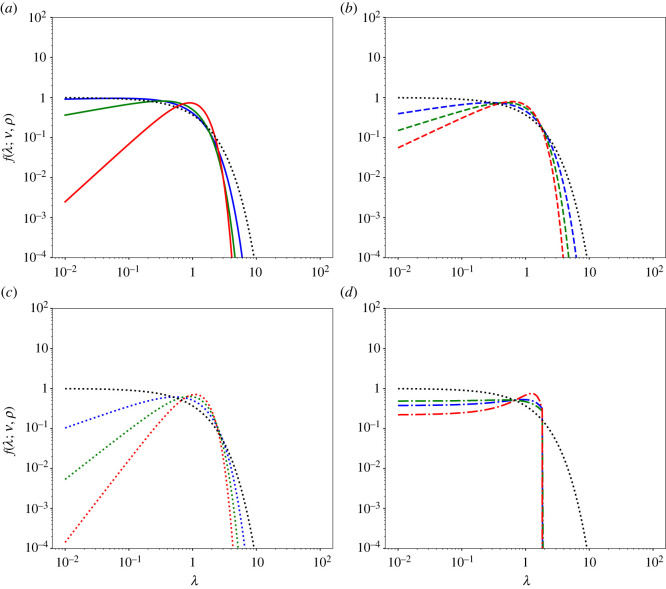
Comparison between the generalized Gamma distribution of the diffusion coefficients *f*(*λ*) (3.3) and the M-function distribution *f*_ggBm_(*λ*) (4.11) for different values of the parameters. (*a*) The generalized Gamma distribution (*ν* = 1.25 and *ρ* = 1.5 (solid blue line), *ν* = 1.5 and *ρ* = 1.75 (solid green line), *ν* = 1.75 and *ρ* = 1.25 (solid red line)). (*b*) The Weibull distribution (*ν* = *ρ* = 1.25 (dashed blue line), *ν* = *ρ* = 1.5 (dashed green line), *ν* = *ρ* = 1.75 (dashed red line)). (*c*) The Gamma distribution (*ρ* = 1) (*ν* = 1.25 (dotted blue line), *ν* = 1.5 (dotted green line), *ν* = 1.75 (dotted red line)). In each panel, the exponential distribution is also displayed (*ρ* = *ν* = 1 (dotted black line)). In all the plots, *λ*_0_ = 1.

**Figure 4.**
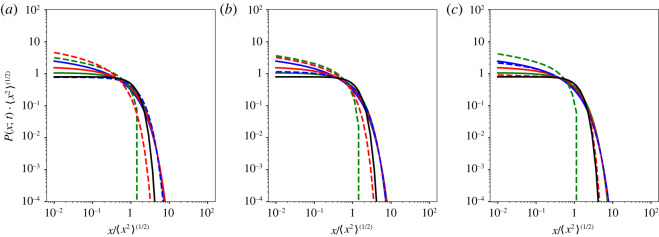
Comparison among the fundamental solutions (3.1), (4.12) and (4.13), i.e. 〈*x*^2^〉^1/2^ *P*(*x*; *t*) vs *x*/〈*x*^2^〉^1/2^, in lin-log scale of the corresponding Fokker–Planck equations (4.5), (4.6), (4.8), (4.10) and (4.9), respectively, with the following values of the parameters: (3.1) with *ρ* = 1.25 and *ν* = 1.5 (solid blue line); (3.1) with *ρ* = *ν* = 1.25 (solid green line); (3.1) with *ρ* = *ν* = 1.5 (solid red line); (3.1) with *ρ* = *ν* = 1/(2*H*) (dashed blue line); (4.12) with *ρ* = *X*1 (dashed green line) and (4.13) (dashed red line). In all the plots, *λ*_0_ = 1. The reference with the Gaussian density is also displayed (solid black line). (*a*) *H* = 0.25, (*b*) *H* = 0.35, and (*c*) *H* = 0.45.

The comparison between the generalized Gamma distribution of the diffusion coefficients *f*(*λ*) (3.3) and the M-function distribution *f*_ggBm_(*λ*) (4.11) is shown in figure 3. Moreover, the comparison among the fundamental solutions (3.1), (4.12) and (4.13) of the corresponding Fokker–Planck equations (4.5, 4.6, 4.8), (4.10) and (4.9), respectively, is shown in figure 4 together with the Gaussian distribution.

## 5. Discussion

In spite of the fact that knowing the solution is the most desirable observable, from the equation, we can indeed obtain remarkable further information on the process. This information can be physical, mathematical and also useful for applications.

In fact, from the physical point of view, we have already observed that the heterogeneity of the diffusion coefficients is the cause of the weak ergodicity breaking, but from the Fokker–Planck equation, we have also information about the relation between physical quantities like, for example, the formula of the flux *q*(*x*, *t*), i.e.

5.1 $$\frac{\partial P}{\partial t}=-\frac{\partial q}{\partial x},$$

that, in the considered case, from the Fokker–Planck equation (4.5), it results as follows [41]:

5.2 $$\begin{array}{rl} q(x,t) & =-2H\lambda_{0}\;t^{2H-1}{\;}_{t}D_{2H\rho }^{\nu /\rho -1,1/\rho }\frac{\partial P}{\partial x}\\ & =-\frac{4H^{2}\;\rho \lambda_{0}\;t^{2H-1}}{\Gamma (n-1/\rho )}\\ & \;\times \frac{\partial }{\partial x}\prod_{\;j=1}^{n}\left[\frac{\nu }{\rho }-1+j+\frac{t}{2H\rho }\frac{\partial }{\partial t}\right]\left\{\frac{\int_{0}^{t}({\left(t^{2H\rho }-\tau^{2H\rho }\right)}^{n-1-1/\rho }/\tau^{1-2H(\nu +1)})P(x;\tau )\;d\tau }{t^{2H[\nu +\rho (n-1)]}}\right\}, \end{array}$$

with *n* − 1 < 1/*ρ* ≤ 1, which is not proportional to the gradient as in standard diffusion and we have indeed that the heterogeneity is the cause of the emergence of a memory kernel. This memory kernel is determined by the specific distribution of the diffusion coefficients *f*(*λ*). Moreover, because of this memory kernel, it follows that the resulting evolution equation is a fractional differential equation, and a special connection exists between the generalized Gamma distribution and the Erdélyi–Kober fractional operators (see [42,53,54]).

Moreover, from the governing equation, we can see if there are forcing terms and their origin and, actually, we have from (4.5) that the heterogeneity of the diffusion coefficient causes a flux with memory, but it does not cause the emerging of any apparent forcing: in fact the resulting governing equation does not include any terms like ∂*F*/∂*x* and preserves indeed the form of the free-particle diffusion equation with the feature of a time-dependent effective diffusion coefficient. In particular, with respect to space, this heterogeneity does not cause any effect and the operator in space remains the second derivative ∂^2^/∂*x*^2^ as in standard diffusion.

Furthermore, the derivation of Fokker–Planck equation (4.5) embodies as a matter of fact a derivation on physical ground of a fractional equation by avoiding the replacement of the operators. Actually, this derivation, together with the derivation of the governing equation of the ggBm [67], is an alternative derivation with respect to that on statistical grounds discussed in literature [42,53,54] on fractional differential equations in the Erdélyi–Kober sense: this physical significance of fractional equations let indeed to overpass questioned issues in fractional-dynamic generalizations and some related constraints that have to be checked [70].

From the mathematical point of view, we observe indeed that the process turns into a process governed by an integro-differential equation, but it remains linear and parabolic, namely, the emergence of a memory kernel is not the cause, for example, of the emergence of a second derivative in time, i.e. ∂^2^*P*/∂*t*^2^, or a finite front velocity, by keeping the main characteristics of the standard diffusion.

To conclude this section on the importance to know the governing equation of a process rather than the solution in a special case, we report that, under the practical point of view and in the direction of applications, to know the equation allows for studying, at least numerically, the solution with particular initial and boundary conditions and also with real settings in complex and multi-dimensional domains, as well as in presence of external forcings.

## 6. Conclusion and future perspectives

In this article, we focused on the signature of anomalous diffusion as measured in many biological systems and in particular as it is measured in the motion of passive tracers inside cytoplasm as the mRNA molecules inside live *E. coli* cells. From the wide literature, we paid special attention to the dataset by Golding and Cox [4] that has been largely analysed (see e.g. [4–11]). Among the many distinctive features, the most important for the present study is the determination of the single-molecule trajectory as a fBm (see e.g. [5,6,19–25]), and also the heterogeneity of the diffusion coefficient according to a Weibull-type distribution that emerges from the data analysis when the observed anomalous behaviour is reproduced through a fBm as the underlying stochastic process [10].

Following this experimental evidence, we slightly generalized the distribution of the diffusion coefficients by using a generalized Gamma distribution in analogy with other similar analysis [18,26,27], we showed that the PDF of the mRNA molecule displacement is related with the Krätzel function, and we derived the corresponding Fokker–Planck equation. The governing equation results to be a fractional diffusion equation in the Erdélyi–Kober sense with a time-dependent effective diffusion coefficient, which cannot be reduced to the governing equations of existing literature models as the CTRW or the ggBm, neither to other models governed by a fractional differential equation with a time-dependent diffusion coefficient.

This last fact is indeed a twofold finding for motivating future investigations on this model setting. In fact, the derived equation (4.5) pushes the research not only towards the mathematical analysis of a novel family of equations as well as towards the development of solid and reliable numerical schemes for studying more realistic systems in multi-dimensional domains with real geometries and general initial and boundary condition but also towards the development of statistical methods and tools for properly taking into account a distribution of diffusion coefficients that can lead to different modelling approaches as those based on an heterogeneous ensemble of particles, e.g. the over- and under-damped ggBm [10,28,29,35–37,48,71], or those based on diffusion in inhomogeneous random environments, e.g. the diffusing diffusivity approach [48,72–75].

Finally, we highlight that the present framework does not include, yet, two quite general and well-established features of anomalous diffusion: the Brownian yet non-Gaussian regime [48,49,72,73,76–78] and the anomalous-to-normal transition [36,76,79,80] that are indeed part of the paradigm of anomalous diffusion [63] and observed in both the continuous-space setting of the diffusion diffusivity models [48,73] and the discrete-space setting of random walks [79] even with solely two Markovian hopping-trap mechanisms [63]. Thus, these last embody the future developments of research on the superstatistical fBm.

## Data Availability

This article has no additional data.
